# Lead Pollution, Demographics, and Environmental Health Risks: The Case of Philadelphia, USA

**DOI:** 10.3390/ijerph18179055

**Published:** 2021-08-27

**Authors:** Michael J. O’Shea, Jonas Toupal, Hasibe Caballero-Gómez, Thomas P. McKeon, Marilyn V. Howarth, Richard Pepino, Reto Gieré

**Affiliations:** 1 Department of Earth and Environmental Science, University of Pennsylvania, Philadelphia, PA 19104, USA; toupal@sas.upenn.edu (J.T.); rpepino@sas.upenn.edu (R.P.); giere@sas.upenn.edu (R.G.); 2 Department of Chemistry, Haverford College, Haverford, PA 19041, USA; hasibegomez@g.ucla.edu; 3 Center of Excellence in Environmental Toxicology, University of Pennsylvania, Philadelphia, PA 19104, USA; mckeont@pennmedicine.upenn.edu (T.P.M.); howarthmv@gmail.com (M.V.H.); 4 Department of Geography and Urban Studies, Temple University, Philadelphia, PA 19122, USA

**Keywords:** Pb pollution, ArcGIS, land use, childhood health risks, environmental health, environmental justice communities, risk assessment

## Abstract

Lead (Pb) soil contamination in urban environments represents a considerable health risk for exposed populations, which often include environmental justice communities. In Philadelphia, Pennsylvania (PA), Pb pollution is a major concern primarily due to extensive historical Pb-smelting/processing activity and legacy use of Pb-based paints and leaded gasoline. The U.S. Environmental Protection Agency (USEPA) organized and/or compiled community-driven soil sampling campaigns to investigate Pb content in surface soils across Philadelphia. Using these data (*n* = 1277), combined with our own dataset (*n* = 1388), we explored the spatial distribution of Pb content in soils across the city using ArcGIS. While assessing Zone Improvement Plan (ZIP)-code level data, we found strong correlations between factors, such as the percentage of children with elevated blood lead levels (% EBLL) and % minority population as well as between % EBLL and % children in poverty. We developed a “*Lead Index*” that took demographics, median measured Pb-in-soil content, and % EBLLs into account to identify ZIP codes in need of further assessment. Our results will be used to help lower the Pb-exposure risk for vulnerable children living in disproportionately burdened communities.

## 1. Introduction

Heavy metal pollution is a significant concern in industrial and post-industrial cities worldwide [1,2,3,4]. Specifically, lead (Pb) pollution is a major environmental and health challenge due to the contaminant’s widespread use mainly during the 19th and 20th centuries for a variety of commercial, industrial, and residential purposes [5,6,7,8]. Common sources of problematic Pb pollution include legacy Pb-based paints, emissions from former Pb-smelting/processing activity, and old leaded gasoline [6,9,10,11,12,13]. Indeed, Pb pollution may be related to factors, such as land use, as well as to traffic density [14] and occurrence of Pb-containing traffic paint (e.g., [15]). However, it is difficult to distinguish between pollutants stemming from human activities and those from pedo-chemical effects, as source apportionment of Pb contamination typically requires detailed isotope analysis and modeling efforts [16,17,18].

Heavy metal pollution has been studied using environmental media, including soils and urban road dust [19,20,21,22,23,24,25,26,27]. Soils and road dusts may record the legacy of an urban environment as the two media act as both source and sink for contaminants [28]. In fact, soil is an understudied medium, which can act as a reservoir for potentially toxic heavy metals such as Pb [29,30]. Lead is persistent in the environment [31] and can exist in soil longer than in other media such as air or water [32]. In the U.S., uncontaminated surface soils have a median Pb-in-soil content of 18 mg/kg, whereas the median in Pennsylvania surface soils, the focus of this study, is 46.4 mg/kg [33,34]. However, urban areas typically have increased Pb-in-soil concentrations compared to other locations (e.g., [35]). The threshold for dangerous levels of Pb in bare soils where children play is 400 mg/kg, according to the United States Environmental Protection Agency (USEPA). Previous investigations have clearly shown correlations between Pb-in-soil contents and blood lead levels (BLLs) [36,37,38,39].

Lead is toxic to most human organs and may act as a neurobehavioral inhibitor, which can impair cognitive development and performance [40,41]. Lead is on the Agency for Toxic Substances and Disease Registry’s (ATSDR) Substance Priority List [42]. Furthermore, the World Health Organization (WHO) has identified Pb as one of ten chemicals of major public health concern (e.g., [43]); as such, action is needed to protect the health of workers, children, and women of reproductive age. Children are at the highest risk from Pb exposure due to high rates of hand-to-mouth contact, their developing brains, and other physiological factors [4,44]. Indeed, this issue is critical as early exposure to Pb can cause substantial cognitive damage, potentially leading to reduced academic and work performance [45], thereby affecting entire communities. Crucially, the Centers for Disease Control and Prevention (CDC) note that there is no level of Pb exposure that is safe [46].

**Figure 1:**
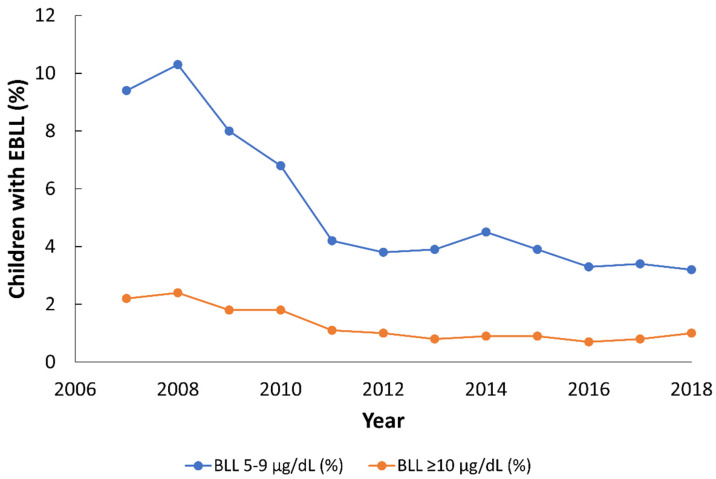
Evolution of the percentage of children with elevated blood lead levels (EBLL), i.e., Pb in blood ≥ 5 µg/dL, from 2007 to 2018. All data are from the city of Philadelphia’s Childhood Lead Poisoning Surveillance Report (2019). The total number of children screened ranged from 35,456 (2014) to 39,146 (2010).

In Philadelphia, the city compiled surveillance data reported to them via health care providers which documented the percentage of elevated blood lead levels (EBLLs), i.e., Pb concentration in blood ≥ 5 µg/dL, in children ages 0 to 6 years [47]. Notably, the percent of children with a BLL of 5–9 µg/dL decreased from 10.3% in 2008 to 3.2% in 2018; similarly, the percent of children with BLL ≥ 10 µg/dL decreased from 2.4% in 2008 to 1.0% in 2018 (Figure 1). Despite these decreases in EBLLs, Pb pollution must still remain a top priority for the city because of the life-long debilitating effects of Pb poisoning (e.g., [39]).

Previous investigations in the United States focusing on Pb pollution demonstrated that correlations existed between EBLL and factors, such as poverty, older housing, and age of population [48,49,50]. Past studies in the United States further demonstrated that Pb contamination was positively related to certain demographic factors: for example, non-Hispanic Black children were found to have the highest % EBLL compared to other races [49,51]. Furthermore, a recent investigation found that Black children are more likely to have EBLLs [52]. Indeed, previous geospatial-based approaches found that socioeconomic status [53,54,55], the year a house was built [56,57,58], and race [59,60,61] were all risk factors that contributed to EBLL. Finally, demolitions [62], for instance those associated with gentrification, must also be considered as potential area-level hazards, because they may resuspend Pb-containing particles deposited in soil. Similarly, demolitions of old buildings will also promote suspension of dust derived from Pb-based paint.

The USEPA and ATSDR collaborated with the University of Pennsylvania on numerous community-based soil screening and health education events in order to support awareness and actions to reduce Pb exposures in the city and to semi-quantitatively identify areas with elevated Pb-in-soil levels. The two goals of the initial campaign were to: (1) determine if home gardeners were being exposed to elevated concentrations of Pb in yard soils; and (2) find if children playing in yards were exposed to elevated Pb-in-soil contamination. In addition, students at the graduate and undergraduate level participated in soil collections through course work and projects. Here we compile the results of these efforts. A past investigation of Pb-in-soil contents in Philadelphia found elevated levels of Pb in soils near former smelter sites compared with residential sites [6]. However, we performed a road dust and soil investigation, which identified legacy Pb paint and leaded gasoline as the main contributors to Pb pollution in the city (e.g., [26]). Another study in Philadelphia found that higher childhood EBLL were strongly related to Pb content in entryway floor dust [63]. Overall, the authors were not able to directly identify a single point source related to childhood EBLL. However, the authors suggest that old housing (built before 1900), legacy Pb paint, legacy polluting facilities, low-income housing, and recent renovation activity all could contribute to the EBLL observed.

The present study sought to explore spatial relationships between Pb-in-soil contents, % EBLLs, and demographic factors. Through the development in this paper of an index tool designed to predict the risk of Pb pollution, we set out to identify areas in need of further evaluation and possible remediation.

## 2. Materials and Methods

### 2.1. Soil Samples

The data set for the surface soil samples came from a variety of sources (see Table S1a,b). We retrieved much of these data from the USEPA following a public records request utilizing the Freedom of Information Act (FOIA) protocol; of the 2665 soil data points used in the present paper, 1277 were compiled by the USEPA and the remaining were from, or in collaboration with, the University of Pennsylvania (Table S1b). The data set partly consisted of information gathered from publicly organized “soil workshops.” These workshops provided the public with the opportunity to bring soil samples to the event to have them analyzed for Pb and other heavy metals using handheld X-ray fluorescence (XRF) spectrometers. These workshops were mainly conducted by the University of Pennsylvania, participating federal agencies, and local community organizations (Table S1b). At these events, citizens generally collected samples from the top 0–15 cm of soils in their yards. Following protocol, residents collected samples in aggregates of five approximately 0.06 L-sized portions (each total sample was approximately 300 mL) from a single area (see Table S1b for details). Subsequent to air-drying and homogenization of the samples, Pb content was determined utilizing the XRF spectrometers. This citizen scientist approach featured broad community participation. Global Positioning System (GPS) coordinates were collected for each sampling site. If the sample was collected from a private residence, the coordinates of the nearest intersection were used to protect privacy. The XRF spectrometers utilized during the various sampling events described included: Innov-X 4000 SL; NITON XLt792YW; Innov-X Delta; Olympus Delta Professional with 40 kV Tube and SDD detector custom configured with modes for soil; Thermo Fisher Scientific XL 3t 600. The detection limits for these instruments ranged from approximately 5–45 mg/kg Pb. These limits depend on the make, model, and age of the XRF and conditions, including soil moisture and calibration capabilities. In some cases, Pb content was confirmed with laboratory-grade inductively coupled plasma-mass spectrometry (ICP-MS) analysis (see Table S1b), which were generally only reported as detectable if concentrations were greater than or equal to 1 mg/kg. A previous study of soils utilized portable XRF spectrometers to determine Pb content and confirmed their XRF results with ICP-MS coupled with an acid digest [62]. A strong agreement (r^2^ = 0.78) was found for the correlation between the results determined by XRF and ICP-MS. Similarly, in a previous investigation, we collected 20 soil samples in the Fishtown neighborhood (ZIP code 19125) of Philadelphia, and analyzed the samples with both XRF and inductively coupled plasma-optical emission spectroscopy (ICP-OES). We found a good agreement between XRF and ICP-OES data (r^2^ = 0.80). The r^2^ value (0.88) was even higher when two outlier contents were omitted.

**Figure 2:**
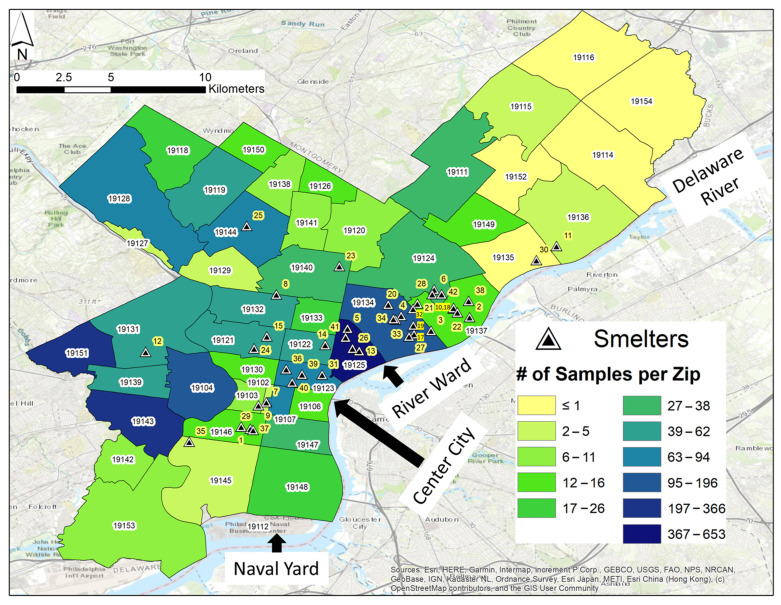
Map of Philadelphia, separated by ZIP codes. The color of each ZIP-code area corresponds to the number of soil sample points. Former smelter/processor sites are referred to above as “Smelters” for simplicity and are labeled as black triangles with white edges and numbered, with numbers highlighted with a yellow halo. Note, sites 10 and 18 overlap and are represented by one triangle. See Table S1a,b for sample site coordinates and view Table S2 for smelter/processor site details. Key points of interest are labeled on the map.

**Figure 3:**
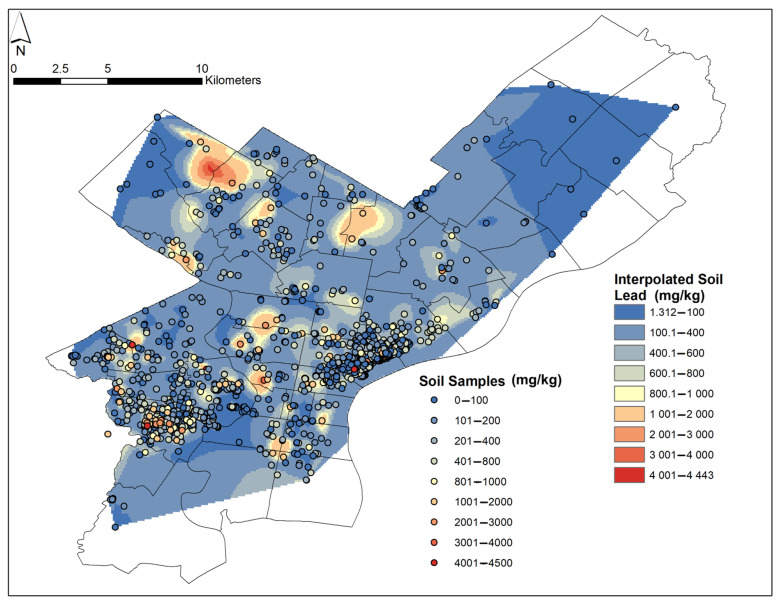
Map of Philadelphia showing all 2665 samples sites with dots showing measured Pb-in-soil content (colored dots). These dots as well as the background layer, which shows interpolated Pb-in-soil content, are color-coded according to Pb content. In the white/blank areas, no samples have been collected, and therefore, we could not provide interpolated Pb-in-soil content there.

Additional sampling was performed, mainly between 2015 and 2020, primarily in West Philadelphia (ZIP codes 19104 and 19143) and the Fishtown area (ZIP code 19125) of Philadelphia, chiefly by students from the University of Pennsylvania (Table S1b). Students were typically enrolled in Richard Pepino’s Urban Pb course, an academically based community service course, which requires students to conduct assignments related to local neighborhoods. Furthermore, soil workshops and sampling events were hosted by The Community Engagement Core of Penn Medicine’s Center of Excellence in Environmental Toxicology (CEET), the only Environmental Health Sciences Core Center to serve this USEPA region (Region 3). Several graduate students mentored by Marilyn Howarth performed sampling to increase the number of included census tracts and to evaluate soils near smelters/processors and schools. The procedure for all University of Pennsylvania sampling programs involved collectors gathering a soil composite of five samples following an “X”-shaped pattern within an area of 1 m^2^ from the top ~1.5 cm of soil. These five samples were then combined, homogenized, and air dried. Subsequently, portable XRF spectrometers were used to determine Pb content. Note that all ZIP codes are labeled in Figure 2 along with sample sites per ZIP code. All sample sites are shown in Figure 3. Note, some past studies have considered surrounding lithology to understand background sources and determine contamination (e.g., [64]). The predominant lithologic unit in the region is the Wissahickon Formation, which consists mainly of garnet-mica schist [65] and is not expected to be a source of Pb.

Limitations: Due to the diverse quality of community data and because of the varied analytical approaches employed, the Pb content data should be used to generally suggest trends and provide health education as opposed to quantitative determinations regarding contamination levels for agency decision making. Other citizen scientist approaches have noted that data must be interpreted carefully and that the quality and limits of data must be accounted for to prevent misrepresentative conclusions [66].

### 2.2. Data Availability

We chose to use Zone Improvement Plan (ZIP) code as the geographical unit of interest because of its familiarity in communications with health care providers and with the general population. Furthermore, ZIP codes facilitated a broad approach focused on general findings. ZIP-code level data for total housing units, % owner-occupied housing units, % rental housing units, median income (in U.S. dollars), % Black population, and % minority population were procured from 2014 GeoLytics Inc. (East Brunswick, NJ, USA) estimates [67]. Note that “total housing units” refers to the number of entire residential buildings, homes, or properties. Each residential building/home/housing unit was counted as one, regardless of the number of rooms or apartments present, and the structures are referred to as properties for simplicity. Similarly, owner-occupied housing units (referred to as % owner-occupied) indicates the percentage of residential properties that owners live in; the definition of housing units is analogous for rentals (referred to as % rented). Geolytics derives its estimates from the U.S. Census Bureau’s data on survey-collected population characteristics. This data set was purchased by CEET and can be found through Geolytics Inc. The percentage of properties built before 1980 (% pre-1980) was obtained from unitedstateszipcodes.org [68]. The data source considers when properties were built to be the year in which the structure was raised; a house or apartment building is one single property. Note that the use of Pb paint was banned in residential settings in 1978 (e.g., [6]). Presumably, the vast majority of homes built after 1978 do not contain Pb paint. Data for % of children with EBLL (% EBLL) and number of demolitions (between 2007 and 2020) [69] were compiled by the City of Philadelphia and were retrieved from OpenDataPhilly. Smelter/processing site data were compiled from the USEPA’s Superfund Program Database [70] and from a key study [71]. The data for % children in poverty were compiled by the research organization Child Trends in 2018 [72]. Data for land use were gathered from OpenDataPhilly [73] where it was uploaded by the Department of Planning and Development.

### 2.3. Geospatial Mapping and Statistical Analyses

The Environmental Systems Research Institute (ESRI)’s geospatial software, ArcGIS (Version 10.5, ESRI, inc., West Redlands, CA, USA) was used to process and map the demographic, % EBLL, and Pb-in-soil content geospatial information used in this study. A choropleth map of the number of soil samples measured per ZIP code, along with point locations of former Pb smelters/processors (Table S2), is shown in Figure 2. The term “measured Pb-in-soil content” is used to refer to Pb-soil values from actual sample locations in this study (see dots in Figure 3). The geographic identifiers for the measured Pb-in-soil content data, collected as either Latitude/Longitude coordinates or nearby street intersections, were geocoded as a point-level layer.

**Figure 4:**
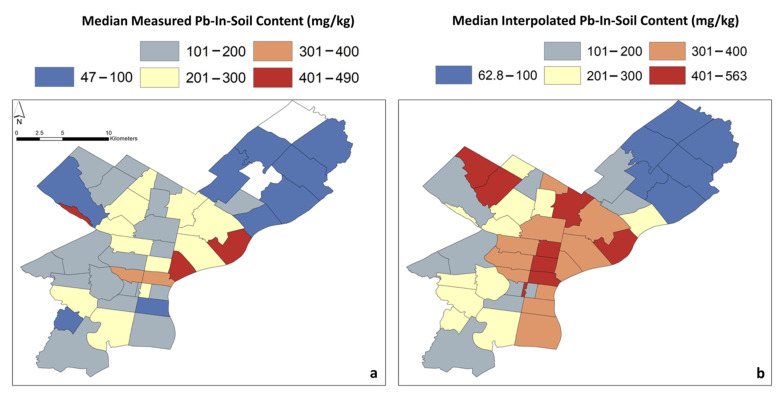
Map of Philadelphia displaying the median measured Pb-in-soil content in each ZIP-code area (**a**) and median interpolated Pb-in-soil content (**b**). The white ZIP-code areas in the left image were the result of zero soil samples with no interpolation taking place.

To approximate Pb-in-soil content in areas surrounding the locations where soil samples were measured, the Natural Neighbor Interpolation tool from ArcGIS’ Spatial Analyst toolbox was applied. The tool finds the closest subset of sample points around each measured site in order to interpolate Pb content (estimates in mg/kg) for the neighboring areas where no measured Pb-in-soil data are available (Figure 3). Below, this is termed “interpolated Pb-in-soil content”. Previous studies have utilized similar techniques in geospatial analysis, such as the well-known inverse distance weighting method [74,75,76,77]. The resultant layer was cropped to the map shape of Philadelphia, PA, using the ZIP-code layer. Next, a map of Pb values was created using two types of Pb-in-soil content for each ZIP code: (a) median values of the measured Pb-in-soil content, referred to below as “median measured Pb-in-soil content”; and (b) median values interpolated from the Pb-in-soil content layer for each ZIP code, termed below “median interpolated Pb-in-soil content” (Figure 4). The interpolated Pb-in-soil content layers were converted from a raster to a point shapefile. Then, the Spatial Join function was used, allowing us to calculate the median for each ZIP code.

**Figure 5:**
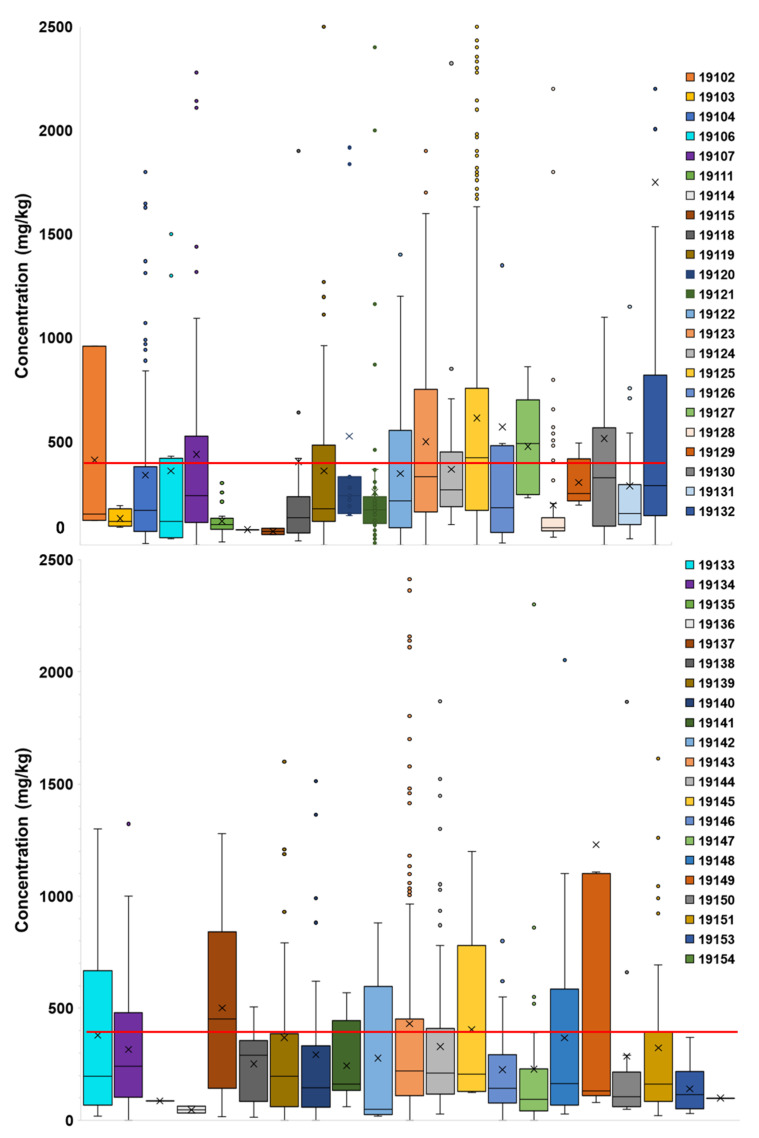
Box and whiskers plots of measured Pb-in-soil contents by ZIP codes in Philadelphia. The box shows the 25–75th percentile, the x is mean content, and the black line is median. Outliers are shown as dots. All graphs were clipped at 2500 mg/kg Pb, excluding outlier points in ZIP codes 19104, 19118, 19125, 19126, 19130, 19131, 19132, 19139, 19143, 19149, and 19151. The red horizontal line denotes the EPA threshold of 400 mg/kg Pb.

Box and whisker plots, using the measured Pb-in-soil contents from the points in Figure 3, were created to display the statistical spread of values by each ZIP code (Figure 5). Spearman rank correlation coefficients (called Spearman correlations herein) were calculated for the factors listed in Section 2.2 and for both median measured (Figure 4a) and median interpolated Pb-in-soil contents (Figure 4b). The correlations were performed in order to statistically determine the relationships between factors. Statistical significance was evaluated at the *p* < 0.05 level at two-tailed significance.

**Figure 6:**
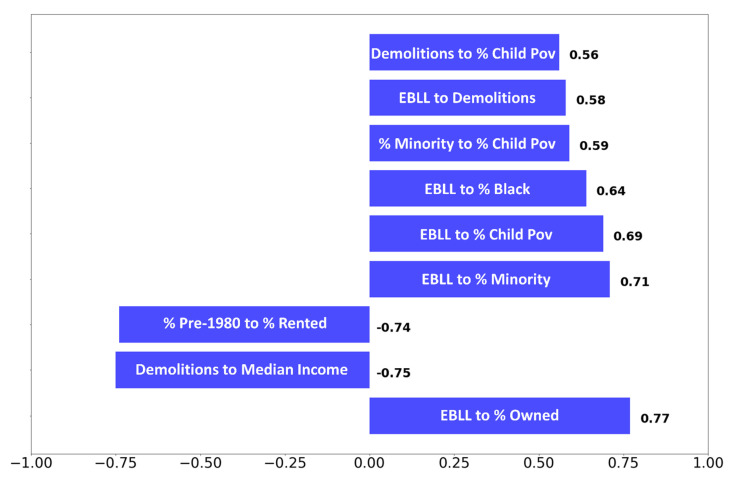
Box plot showing the strongest Spearman correlations present in the data set. Abbreviations: % Child Pov = % children in poverty; % Minority = % minority population; % Black = % Black population; % Owned = % owner-occupied properties; EBLL = % children with EBLL.

**Figure 7:**
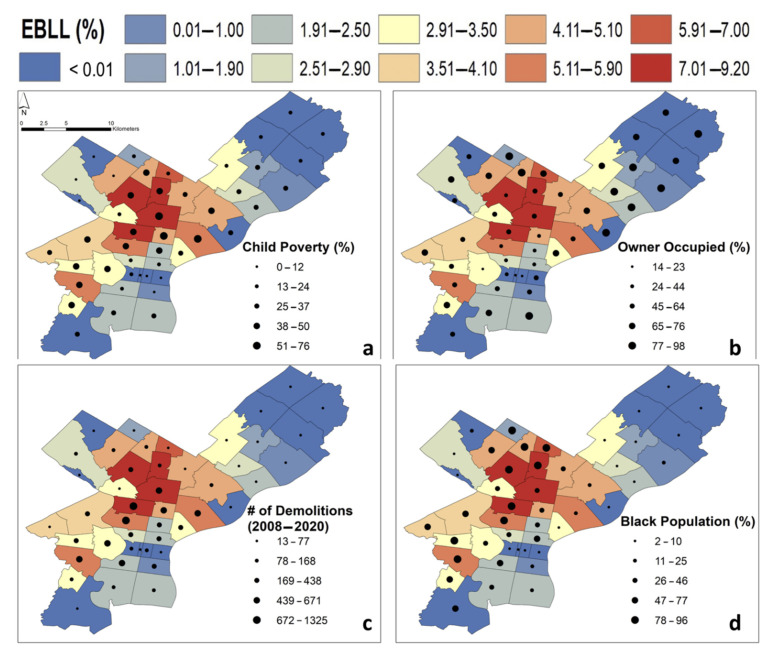
Bivariate plots demonstrating % EBLL compared with % children in poverty (dots) (**a**); % EBLL compared with % owner-occupied properties (dots) (**b**); % EBLL compared with the number of demolitions (dots) (**c**); and % EBLL compared with % Black population (dots) (**d**).

**Figure 8:**
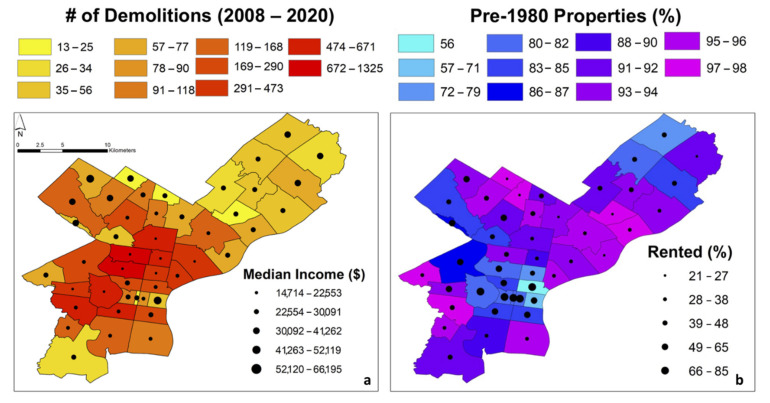
Bivariate plots displaying number of demolitions compared with median income (dots) (**a**), and % pre-1980 properties compared with % rented properties (**b**).

Maps were created to display potential demographic risk factors by ZIP code. The factors presented were chosen due to their high Spearman correlations (Figure 6). A complete listing of Spearman correlations is shown in Table S3. For these bivariate maps, graduated symbols were plotted to represent the second variable (Figure 7 and Figure 8).

**Figure 9:**
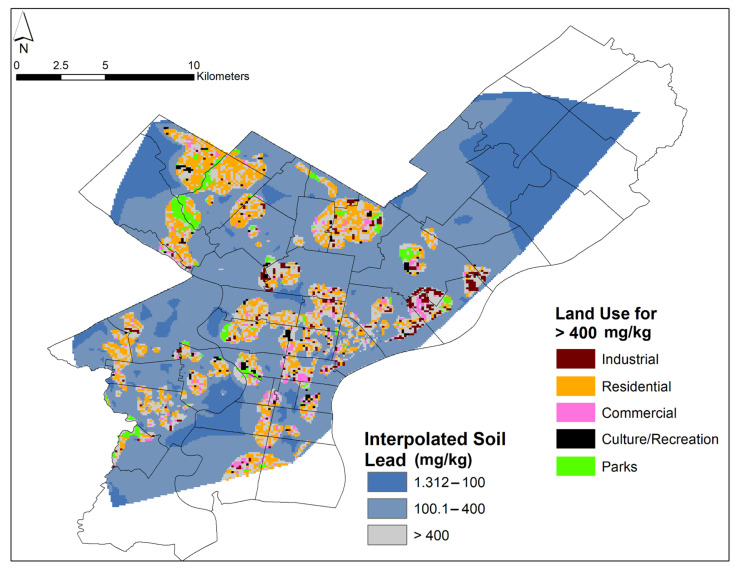
Map of Philadelphia displaying the interpolated Pb-in-soil contents grouped by land use. Areas with Pb ≥ 400 mg/kg are color-coded for their corresponding land use (industrial, parks, residential, culture/recreation, and commercial). Grey areas with Pb ≥ 400 mg/kg correspond to a type of land use not included in this study.

Another map was created that combined the interpolated Pb-in-soil content map with land use (termed “interpolated Pb-in-soil contents grouped by land use”, below, for clarity) (Figure 9). Common land uses (industrial, parks, residential, culture/recreation, and commercial) were displayed for areas with soils that were rich in Pb (≥400 mg/kg). The goal of this visualization was to see which land uses may be related to Pb-rich soils. Note that, for all maps, ZIP code 19112 was omitted for clarity as the area primarily encompasses the Philadelphia Naval Yard (see Figure 2). The ZIP code is mostly unpopulated and thus, population data are not available for it; no soil sampling was performed in 19112, and the ZIP code is not discussed in this manuscript.

### 2.4. Lead Index

To quantitatively predict high-risk areas, we created a “*Lead Index*”, which took into account key factors selected due to their strong Spearman correlations. These factors included: % owner-occupied properties (coded as “Owner”), % pre-1980 properties (Pre1980), number of demolitions (Demos), % minority population (MinorPop), % children in poverty (ChildPov), median income in USD (MedIncome), % children with EBLL (% EBLL), and median measured Pb-in-soil content in mg/kg (MedMeasLead). The factors utilized were based on statistical analysis of available data but could be adjusted in future approaches. To account for differences in perceived importance between factors, those with the highest potential risk were weighted more strongly. The weight of a specific factor *i* (*W_i_*) was qualitatively assigned based on the potential risk of the factor. When determining weight, we considered which factors had the highest Spearman correlations, for example with % EBLL, and information from previous studies [52,54,59]. We sorted the factors into three weights (1, 2, and 3). We assigned the most weight (3) to % EBLL and MedMeasLead due to their importance in indicating Pb pollution. The next set of key factors with high Spearman correlations (MinorPop; ChildPov; MedIncome) were assigned a weight of 2, and the remaining factors were assigned a weight of 1 due to their smaller Spearman correlations. Future studies can change the weight and formulation of each factor to represent their researchers’ interpretation of risk. 

The following Equation (1) was used to calculate the points (*Y*) for each factor (*i*).

(1) $$Yi=\frac{W_{i}}{\left({\bar{x}}_{i}+\sigma_{n}\right)}\times V$$

In the above equation, the weight of the factor assigned (*W_i_*) was divided by the mean of all values of the factor ($\bar{x}$_i_) plus one standard deviation of this mean ($\sigma_{n}$); the term was then multiplied by the value (*V*) of the factor per ZIP code.

As an example, the mean (3%) of the entire dataset for the factor % EBLL plus one standard deviation (2.6%) was 5.6%. The overall factor is weighted as 3. Thus, should a ZIP code have a value of 7% EBLL, it would count as (3/(3 + 2.6%) × 7%) or 3.8 points. Equation (1) was used to calculate the points for each factor (*Yi*), and then the factors were summed, as demonstrated in Equation (2).

Note, median income is the only negative value, as we predict places with lower median income to be more contaminated with Pb.

The *Lead Index* was calculated using the Raster Calculator in ArcGIS for each ZIP-code area. Units are described above but are not in Equation (2) for clarity. The exact equation, with the chosen factors in quotation marks, is as follows:

(2) $$\mathrm{Lead}\;\mathrm{Index}=\frac{1}{62+19}\times “\mathrm{Owner}”+\frac{1}{89+8}\times “\mathrm{Pre}1980”+\frac{1}{\begin{array}{c} 238+301 \end{array}}“\mathrm{Demos}”+\frac{2}{52+30}\times “\mathrm{MinorPop}”\;+\frac{2}{30+17}\times “\mathrm{ChildPov}”+(-\frac{2}{34030+10770}\times “\mathrm{MedIncome}”)+\frac{3}{3+2.6}\times “\%\;\mathrm{EBLL}”+\frac{3}{\begin{array}{c} 188+101 \end{array}}\times “\mathrm{MedMeasLead}”$$

## 3. Results

Former Pb smelter/processor sites are highlighted in Figure 2, with site information described in Table S2. The ZIP-code areas where former smelter/processor sites were mainly concentrated include: 19123, 19125, 19134, 19137, and 19146. Three of these areas are located in the River Ward of the city near the Delaware River, whereas ZIP codes 19123 and 19146 are in Center City. Figure 2 displays the number of measured soil samples per zip code. Lesser measured ZIP codes are all in Northeast Philadelphia and were not targeted during the sampling campaigns. Those ZIP codes were not measured as much due to the higher median income, lower % minority populations, fewer demolitions, and thus, lower potential pollution risk.

The results for the interpolated Pb-in-soil contents, illustrated in Figure 3, demonstrated localized high values in areas, such as Southwest Philadelphia (ZIP codes 19104, 19143) and Northwest Philadelphia (ZIP codes 19118, 19119, 19144). However, several of the areas with high interpolated Pb-in-soil contents were related to just a few points (Figure 3).

When the median measured Pb-in-soil contents were considered, the ZIP codes with the highest contents were 19123 (331 mg/kg), 19125 (421 mg/kg), 19127 (490 mg/kg), 19130 (325 mg/kg), and 19137 (452 mg/kg) (Figure 4a). The ZIP codes with the highest median interpolated Pb-in-soil contents (Figure 4b), on the other hand, were 19102 (563 mg/kg), 19118 (498 mg/kg), 19119 (497 mg/kg), 19120 (420 mg/kg), 19122 (415 mg/kg), 19123 (416 mg/kg), 19133 (471 mg/kg), and 19137 (445 mg/kg).

Box and whisker plots of measured Pb-in-soil contents for each ZIP code (Figure 5) helped to emphasize the variation between mean and median Pb-in-soil contents. The ZIP codes with mean measured Pb-in-soil contents greater than 400 mg/kg Pb were: 19102, 19107, 19123, 19125, 19126, 19127, 19130, 19132, 19137, 19143, 19145, and 19149 (see Figure 5).

Of all factors tested, the strongest statistically significant (*p* < 0.05) Spearman correlations across the entire data set were between: % EBLL and the % owner-occupied properties (0.77); the number of demolitions and the median income (−0.75); the % pre-1980 properties and the % rented properties (−0.74); % EBLL and the % minority population (0.71); % EBLL and the % children in poverty (0.69); % EBLL and the % Black population (0.64); the % minority population and the % children in poverty (0.59); % EBLL and the number of demolitions (0.58); and the number of demolitions and the % children in poverty (0.56) (Figure 6).

In Figure 7, we present % EBLL as the base for all four maps and demonstrate that the highest levels are present in North Philadelphia.

Figure 7 displays bivariate maps showing factors that, when correlated with the base of % EBLL, have among the strongest r values across the entire data set (Figure 6). The ZIP codes where these factors were the most strongly correlated, in almost all cases, were in North Philadelphia (e.g., 19132, 19140, 19141, and 19144).

Similarly, Figure 8 shows bivariate maps highlighting relationships between other factors. Specifically, we display factors that have very strong, albeit negative r values (Figure 6), i.e., the number of demolitions compared with median income (Figure 8a), and the % pre-1980 properties compared with the % rented properties (Figure 8b). The ZIP codes 19104, 19121, 19132, 19133, and 19140 are characterized by a low median income as well as a greater number of demolitions (Figure 8a). However, the greatest number of demolitions primarily were in North and Southwest Philadelphia, closer to Center City. A high % pre-1980 properties, i.e., those that are most likely to have had Pb-based paint applied, were found in North and Southwest Philadelphia (e.g., ZIP codes 19138, 19143, 19150) (Figure 8b).

Figure 9 highlights areas with high interpolated Pb-in-soil contents grouped by land use. Of the land uses analyzed, residential areas were most commonly polluted by Pb and hotspot locations were found in Center City, North, and West Philadelphia (e.g., ZIP codes 19118, 19119, 19120, 19121, 19128, 19132, 19143, and 19151). A cluster of industrial areas with interpolated Pb-in-soil contents grouped by land use greater than or equal to 400 mg/kg were found in the River Ward area (ZIP codes 19132, 19137). Soil samples in some park locations also had high interpolated Pb-in-soil contents grouped by land use ≥400 mg/kg (e.g., in ZIP codes 19119, 19121, 19124, and 19128).

**Figure 10:**
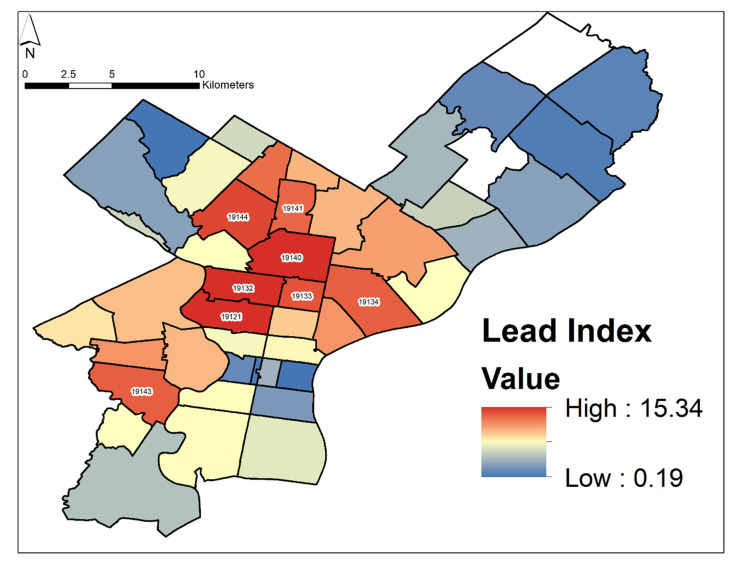
Calculated *Lead Index*—see the Methods section for full details on the model inputs. The ZIP codes with the highest values are labeled (19121, 19132, 19133, 19134, 19140, 19141, 19143, 19144).

The results of our *Lead Index* calculation in Philadelphia, as defined by the key risk factors (see Methods section) and median measured Pb-in-soil content, predicted several areas of potential elevated Pb exposure risk for citizens. The highest-risk ZIP codes were: 19121, 19132, 19133, 19134, 19140, 19141, 19143, and 19144 (Figure 10). The areas with the highest *Lead Index* values are located primarily in North Philadelphia in ZIP codes that are characterized by a predominantly low median income, a high % minority population, and high % EBLL incidences, but where fewer soil samples were measured.

## 4. Discussion

One of the goals of community-focused citizen science is to provide data and information to policy makers in order to identify risks that are present in the local environment and warrant further investigations. In addition, the practice of community-based citizen science helps to engage residents in neighborhoods about the presence of hazardous materials and provides them with data that they can use to improve their environmental health. Through our combined approach, we utilized soil workshop data from the public in combination with data collected by the USEPA and students to holistically explore the Pb-in-soil contents across Philadelphia (Figure 2).

A recent manuscript outlined potential pathways to reduce global disease burdens on populations and highlighted the prominent role that citizen science can play to assist communities that lack resources [78]. These authors argued that citizen science is a tool that empowers people with data about their own environment in order to rectify pollution issues. Filippelli and coworkers led soil sampling programs through Indiana University-Purdue University Indianapolis and Macquarie University, which resulted in >15,000 soil samples collected. Scientists created *mapmyenvironment.com* (accessed on 9 September 2020) [79], a hub for this information where users can upload soil data [80]. Ultimately, these efforts highlight the importance of working with communities to collect and visualize data. We plan to continue to collect and map soil data in Philadelphia through citizen science (*https://ceet.upenn.edu/leadsoilmap/* (accessed on 1 January 2021)) [81] and use these data to enhance environmental health literacy about Pb and its effects on human health and impact policy to protect residents from Pb exposure. Citizen science is also used to promote regulatory agencies’ interest and activity in environmental justice communities that might otherwise fall between the cracks.

In the current study, one shortcoming to be addressed is the uneven sampling across the study area. Some ZIP codes (e.g., 19125, 19143, and 19151) had upwards of 190 samples each, whereas others (e.g., 19114, 19115, 19116, 19135, 19152, and 19154) had fewer than five samples per ZIP code (Figure 2). This sampling disparity was a result of soil sampling events that largely focused on known areas of former industrial activities, such as Fishtown (ZIP code 19125) and Port Richmond (ZIP code 19125 and 19134). Similarly, areas close to the University of Pennsylvania campus that were known to have Pb-contaminated soils, such as West Philadelphia (ZIP codes 19104, 19143), were targeted. Therefore, when the map showing the interpolated Pb-in-soil content was generated (Figure 3), some hotspot areas were driven by only a few data points (e.g., parts of Northwest and North Philadelphia). Furthermore, this uneven sampling must be considered when comparing ZIP-code level demographic data with data for interpolated Pb-in-soil content. Therefore, the results of our inquiry are general and should not be used to fully characterize hazards. Other studies have utilized gridded approaches in order to combat sampling bias [82]. However, gridded sampling may inhibit community involvement due to the strict sampling needs.

Another important limitation of our study is that we only measured total Pb concentrations, thus neglecting the issue of the bioavailability of Pb. Indeed, the bioavailability of Pb depends on factors, such as the Pb phase present (as previously explored in Philadelphia soils [23]), the characteristics of soil, and diet (e.g., [83]). Past research on rats, for example, has shown that 15–85% of the total Pb-in-soil was bioavailable [84]. Despite this limitation, previous work has shown that total and bioavailable Pb can be highly correlated (e.g., [82]), and thus, studying total Pb is still informative for prediction of risks to communities.

Researchers intending to use XRF for screening Pb-in-soil to identify urban areas with elevated contamination should consider instituting more rigorous protocols such as sieving all soil samples to a health-relevant size fraction, confirming all XRF results with multiple measurements and analytical techniques, and oven drying soil samples. However, these measures may prove impracticable for soil sampling events focused on community health education regarding Pb-exposure prevention.

The ZIP codes with the highest median measured Pb-in-soil contents were 19123 (Center City), 19125 (River Ward), 19127 (Northwest Philadelphia), 19130 (Center City), and 19137 (River Ward) (Figure 4a). The locations with high median measured Pb-in-soil contents included areas of former smelter/processor sites in the River Wards (Figure 1 and Figure 4).

Areas with high median interpolated Pb-in-soil contents included much of Center City and parts of Northwest and Northeast Philadelphia (e.g., ZIP codes 19118, 19119, 19120, 19122, 19123, 19133, 19137) (Figure 4b).

There was significant variability in measured Pb-in-soil contents by ZIP code. It is clear from the general distributions of data (Figure 5) that many ZIP codes (e.g., 19104, 19118, 19125, 19126, 19130, 19132, 19131, 19139, 19143, 19149, and 19151) featured outlier sites that drove mean measured Pb-in-soil contents well above those of the median (Figure 5). It is of note that eight ZIP codes) have a median interpolated Pb-in-soil content above 400 mg/kg Pb (Figure 4b). While the federal threshold for contaminated bare soils where children play is 400 mg/kg, in some specific states, the limits are lower. For example, Maryland recently implemented a tiered approach for soil screenings, where the residential limit is 200 mg/kg Pb, but the commercial and industrial soil thresholds are higher (550 mg/kg and 1050 mg/kg, respectively) [85]. Maryland’s approach was implemented to bring soil screenings in line with the current knowledge of soils’ contribution to EBLL. The state of California has even stricter standards, with a threshold of 80 mg/kg Pb for residential/unrestricted land use and 320 mg/kg for commercial/industrial land use [86]. Almost all Philadelphia ZIP codes (40 out of the 44 considered) feature median interpolated Pb-in-soil content greater than 80 mg/kg, except for ZIP codes 19114, 19115, 19136 and 19152, which are all located in Northeast Philadelphia (see Figure 4b).

As previously noted, many of the strongest correlations were observed between % EBLL and other factors, such as the % owner-occupied properties (0.77); the % minority population (0.71); the % children in poverty (0.69); and the % Black population (0.64) (Figure 6). Furthermore, the number of demolitions was significantly (<0.05) related to % EBLL (0.58) and also strongly negatively (−0.75) correlated to median income. The resuspension of soils with high Pb contents, potentially associated with gentrification activities, may explain the link between number of demolitions and % EBLL. Specifically, the Pb-in-soil contents determined in Fishtown could be partially related to the recent gentrification that has occurred within this area. A recent investigation of Pb-in-soil contents in New York City found that parks and greenspaces in redeveloping areas with high population growth had higher levels of Pb-in-soil contents compared with other land uses [62]. The authors speculated that the high Pb-in-soil contents could also occur in residential areas. Indeed, our investigation of interpolated Pb-in-soil contents grouped by land use (≥400 mg/kg, Figure 9) demonstrated that residential areas may be highly contaminated (e.g., as observed in ZIP codes 19118, 19119, 19120, 19121, 19128, 19132, 19143, and 19151). Pavilonis and coauthors suggested that new construction that could disrupt existing soils and the demolition of older buildings (presumably coated with Pb paint) in historically contaminated areas could account for the high levels Pb-in-soil contents in the nearby parks and greenspaces. Previous studies established that areas with older housing were correlated with higher Pb-in-soil contents [87,88]. Zoning policy may also be an important contributor to these findings, as suggested by the data shown Figure 9: this figure reveals industrial land use in close proximity to residential use in most areas of high Pb-in-soil values. This close proximity of residential and industrial properties is likely due to the fact that zoning in Philadelphia has historically been on a per lot basis at the ‘Councilmember’s Prerogative’ rather than in districts with similar land use [89]. Historic and current land use may be discordant and pose a risk of exposure to legacy Pb pollution.

Using the Spearman correlations, we created a visualization of factors that were strongly correlated, such as % EBLL compared with % children in poverty (Figure 7a); the ZIP codes where both factors had high values included 19121, 19132, 19140, 19141, 19143, and 19144. The same ZIP codes, in addition to 19126, featured high % EBLL values and a high % of minority and Black populations (Figure 7d). The strong correlations between % EBLL values and % of minority and Black populations point to a race-related public health issue. When % EBLLs were compared with the number of demolitions (Figure 7c), similar ZIP codes had high values for both factors (e.g., 19121, 19132, 19140, and 19143). In Figure 8a, the number of demolitions was negatively correlated to median income (see also Figure 6). Therefore, the socioeconomic aspect of EBLL should be further studied as well.

Another important statistic is the % pre-1980 properties, as they may still contain legacy Pb paint. When % pre-1980 properties were compared with the % rented properties, the correlation was strong and negative (−0.74), which could mean that newer properties may be preferentially rented (e.g., newly constructed apartments in Center City; see Figure 8b). On the other hand, there is a strong positive correlation between % EBLL and % owner-occupied properties (0.77). This correlation is unique to Philadelphia as the city has high rates of home ownership due to affordable row homes stemming from past housing policies [90]. The end result is that many relatively poor families own rather than rent their homes [91]. Therefore, while rental properties are usually a risk factor due to having more health code and housing violations (e.g., [90]), in Philadelphia, many homeowners are also at risk. Overall, areas in need of special consideration due to the factors outlined above include parts of North and West Philadelphia (Figure 7 and Figure 8). There is a significant overlap between areas with high median measured Pb-in-soil content and areas with high risk factors.

The holistic approach utilized in this study included an analysis of land use (Figure 9). We found that areas with elevated interpolated Pb-in-soil contents grouped by land use were primarily residential. Indeed, residential interpolated Pb-in-soil contents grouped by land use may be higher than those in soils in public spaces. This result is in part due to the different management structures: residential areas are unlikely to be managed by the city, as opposed to land with other uses. Pockets of high interpolated Pb-in-soil contents grouped by land use were also found in industrial areas near former smelter/processor sites in the River Wards of Philadelphia (e.g., ZIP codes 19132, 19137); similarly, some parks in Northern Philadelphia feature high interpolated Pb-in-soil contents grouped by land use (e.g., in ZIP codes 19119, 19120, 19121, 19124, 19128). The pollution in parks should be explored as families with children often frequent these places. Overall, the dominance of residential sites, primarily in North and West Philadelphia, point to areas that require more sampling.

The *Lead Index* represents a step forward in advancing a quantitative approach to identifying ZIP codes of potentially high risk using combined environmental, demographic, and % EBLL data. The identified high-risk areas include ZIP codes 19121, 19132, 19133, 19134, 19140, 19141, 19143, and 19144, which are mainly in North Philadelphia. The index has similarities to the previously established Lead Risk Index, developed by the National Minority Quality Forum [92]. The Lead Risk Index utilized data from the 2005–2010 National Health and Nutrition Examination Survey (NHANES) and the CDC’s state surveillance data on blood Pb tests. The index was built on Bayesian hierarchical regression models to assess risk factors including gender, race/ethnicity, age, poverty status, old housing stock, and blood Pb data. However, the previous model lacks data for median measured Pb-in-soil content, whereas our model lacks data on gender.

A recent investigation on potential soil-Pb exposure at the household scale in Greensboro, North Carolina, combined sampling, statistical analysis, and machine learning and revealed racial disparities in Pb-in-soil concentrations [93]. Indeed, these results reinforce the importance of using our index or a similar method to guide exposure prevention efforts.

A similar study [59] modeled EBLL, housing, and socioeconomic data at the ZIP-code level throughout all of New York State and found that EBLLs were correlated to older housing, to lower proportions of high school graduates, and to births to African American mothers. The authors recommended a combination of educational and remediation programs targeting areas identified as high risk, and that these communities should be further assessed in detail. Similarly, we recommend that similar measures be taken in Philadelphia, with priority for the highest-risk ZIP codes (19121, 19132, 19133, 19134, 19140, 19141, 19143, and 19144).

Another lens through which to analyze the *Lead Index* is to use historic redlining maps. These were discriminatory maps created by the Home Owners’ Loan Corporation, primarily in the 1920s and 1930s, which were used to determine who received loans [48]. Areas with Black populations were marked as “hazardous”. While these racist practices were later abolished, their impact may still be felt today [48]. When Figure 10 was compared with a 1937 redlining map created by the Home Owners’ Loan Corporation, there was some overlap between “hazardous” areas and places with higher values of *Lead Index* developed here (e.g., ZIP codes 19104, 19121, 19125, and 19134) [94]. However, other areas marked as “hazardous” in the redlining map include most of Center City, which in our study did not show high *Lead Index* values. Similarly, areas in North Philadelphia that were at risk according to the *Lead Index* were not shown as “hazardous” on the redlining map, indicating disparity between the two metrics. Though, the changes to the city from 1937 to present day must also be considered. For example, Center City today has many brand-new high-rises with little Pb hazard, whereas this was not the case in 1937. Overall, redlining maps should be explored in future studies to better understand the historic context of Pb pollution.

In terms of data sets, a research group performing analysis in the state of Michigan demonstrated that assessing census-block groups can better predict variance in BLL data than census-tract or ZIP-code variables [54]. The authors found that incorporating risk factors, such as housing built before 1940, socioeconomic status, and racial/ethnic characteristics were key in explaining BLL variance. However, other studies in Massachusetts and Rhode Island found that census tracts were more predictive for health outcomes than ZIP-code or census-block data [95]. Indeed, a recent investigation [96] examined BLL using census-tract data in Milwaukee County and found that low home-ownership, high-poverty, and non-white census tracts contributed to higher BLLs compared with the opposite factors. They argued that Pb-poisoning prevention begins with equity in housing and economic policies designed to assist Pb-burdened communities. To provide a more detailed approach that complements the broad assessment presented in our study, census-tract data were analyzed in the high-risk areas highlighted here [91]. Overall, through the census-tract investigation, the specific sources of Pb identified and the factors that contributed to EBLL were in agreement with the current work.

Other investigations, such as that by the Michigan Department of Community Health, relied on dichotomized ZIP-code data [54]. However, ZIP-code boundaries can change through time [97]. Therefore, our approach simply represents a broad examination in Philadelphia designed to advance the conversation, narrow the focus and point to areas of concern while combining diverse data sets. The major difference between our investigation and the previously mentioned reports is that the latter did not consider median measured Pb-in-soil content and were focused on BLL data related to demographic information, whereas our study seeks to incorporate environmental risk by specifically including geochemical data as well.

A previous investigation [56] in New Orleans and Lafourche Parish, Louisiana reached similar conclusions to the current study and demonstrated that soils must be a primary consideration for Pb-poisoning prevention.

## 5. Conclusions

This study represents an important step in further developing Pb-pollution investigations in Philadelphia. Moreover, it provides a new tool to assess Pb-pollution hazards, which represent an ongoing public health risk to the most vulnerable members of the community, not just in Philadelphia but also in other older urban settings. The authors hope that future investigations can refine the tools used and developed here, such as the *Lead Index*, to ultimately improve predictive models of high-risk areas.

Strong positive correlations between % EBLL and % minority populations; % EBLL and % children in poverty; and % EBLL and % Black population were found. These concerning results highlight that Pb pollution is not only an environmental health problem but also an environmental justice issue. Going forward, Pb contamination must be analyzed as a race-related public health issue and, as past studies have suggested, prevention of Pb poisoning requires that economic policies and Pb education programs be created to aid Pb-burdened communities.

We used environmental, demographic, and % EBLL data to create a *Lead Index*, which identified ZIP codes (19121, 19132, 19133, 19134, 19140, 19141, 19143, and 19144), mainly in North and West Philadelphia, that host Pb-burdened communities in the city and thus must receive highest priority for additional sampling and/or Pb-mitigation measures.

Ultimately, our study represents a broad effort to create a model that brings together many stakeholders including citizens, students, regulatory agencies, and even the media.

## Data Availability

Data are contained within the Supplementary Materials. All other data is available as described in the Methods section of the paper.

## Supplementary Materials

The following are available online at www.mdpi.com/article/10.3390/ijerph18179055/s1, Table S1a: Sample sites and Pb-in-soil content: Table S1b: Sources of sample data, Table S2: Lead Smelter/Processor Sites, Table S3: All Spearman correlations.
